# Expert consensus from the Chinese medical association on pharmaceutical management of combined cardio-oncology physician-pharmacist clinics

**DOI:** 10.3389/fonc.2026.1741387

**Published:** 2026-06-17

**Authors:** Ziyan Lyu, Hui Huang, Yuan Bian, Yue Zhang, Xisha Xue, Min Chen

**Affiliations:** 1 Department of Pharmacy, Sichuan Academy of Medical Sciences & Sichuan Provincial People’s Hospital, Chengdu, China; 2 Department of Pharmacy, Personalized Drug Research and Therapy Key Laboratory of Sichuan Province, Sichuan Provincial People’s Hospital, School of Medicine, University of Electronic Science and Technology of China, Chengdu, China; 3 Department of Cardiology, Sichuan Academy of Medical Sciences & Sichuan Provincial People’s Hospital, Chengdu, China

**Keywords:** cardio - oncology, cardiotoxicity, combined physician - pharmacist clinic, expert consensus, pharmaceutical management

## 1 Introduction

Data released by the International Agency for Research on Cancer (IARC) of the World Health Organization reveals that in 2020, there were 19.29 million newly diagnosed cancer cases globally, among which 4.57 million occurred in China. Projections indicate that this figure is expected to reach 28.40 million by 2040 (1). The complexity of cancer - related cardiovascular diseases poses new challenges for medical practitioners, and overcoming the limitations of single - discipline approaches has become an urgent issue in cardio - oncology specialty clinics. Currently, cardiotoxicity caused by anticancer drugs is a major factor contributing to mortality among cancer patients (2). The growing focus on cancer - related cardiovascular diseases has given rise to the interdisciplinary field of cardio - oncology. Cardio - oncology encompasses the study of cancer patients with pre - existing cardiovascular conditions and cardiovascular complications induced by anticancer therapies (3). For example, anthracyclines can induce myocardial injury through enzyme - mediated and non - enzyme - mediated generation of oxygen free radicals (4), whereas immune checkpoint inhibitors may trigger immune - mediated myocarditis via T - cell activation (5).

To enhance pharmaceutical administration and services in medical institutions, elevate management levels, facilitate rational drug utilization, and more effectively safeguard public health, the National Health Commission, in conjunction with the National Healthcare Security Administration and the National Health Commission of China, in conjunction with the National Healthcare Security Administration, the Ministry of Education, the Ministry of Finance, the Ministry of Human Resources and Social Security, and the National Medical Products Administration in China, issued the Notice on Printing and Distributing the Opinions on Strengthening Pharmaceutical Administration in Medical Institutions to Promote Rational Drug Use (National Health Commission Medical Administration (2020) No. 2).This directive stipulates that medical institutions shall strengthen drug inventory management, promote the rational utilization of medications, and broaden the scope of pharmaceutical services. Specifically, it advocates the establishment of pharmacist-led clinics to offer medication counseling and guidance. As a result, specialized pharmaceutical clinics have witnessed a rapid expansion across diverse medical institutions. In March 2022, Sichuan Provincial People’s Hospital established a combined cardio - oncology physician - pharmacist clinic, staffed by cardiologists and clinical pharmacists, to guarantee the rational use of drugs in cancer patients.

In view of the absence of established management systems and diagnostic workflows for such clinics, Sichuan Provincial People’s Hospital took the initiative, under the sponsorship of the Clinical Pharmacy Branch of the Chinese Medical Association, to solicit opinions from national experts in the fields of clinical pharmacy, medical oncology, cardiology, and evidence - based pharmacy. By referring to domestic and international guidelines and research on cardio - oncology, and based on regulations such as the Pharmaceutical Administration Regulations of Healthcare Institutions (6), Pharmaceutical Service Standards of Healthcare Institutions (7), and Prescription Review Standards of Healthcare Institutions (8), as well as the practical experiences of multiple tertiary hospitals including Sichuan Provincial People’s Hospital, this Expert Consensus on Pharmaceutical Management of Combined Cardio - Oncology Physician - Pharmacist Clinics (hereinafter referred to as the “Consensus”) was formulated. Its aim is to standardize the management of combined clinics and enhance the effectiveness, safety, and economy of pharmacotherapy.

## 2 Consensus development process

### 2.1 Composition and responsibilities

The Consensus drafting committee was composed of experts and scholars from clinical pharmacy, medical oncology, cardiology, and evidence-based pharmacy. It included a chair, review experts, writing experts, and secretaries.

### 2.2 Registration

The Consensus has been registered on the International Practice Guideline Registry Platform (http://www.guidelines-registry.org) (under Registration No. PREPARE-2022CN772).

### 2.3 Development steps and methods

#### 2.3.1 Selection and finalization of clinical questions

The drafting committee initially recognized pharmaceutical management issues in four domains, comprising 11 clinical queries, via a systematic retrieval of published guidelines, systematic reviews, and relevant research in cardio - oncology. This was complemented by interviews with physicians and pharmacists in this field. The Delphi technique was utilized to conduct a survey and assign scores to the significance of these queries. Consensus was delineated as a full - score ratio >exceeding 50%, a mean score greater than 3.5, and a coefficient of variation less than 0.25. Following two rounds of online consultations with 38 experts, 11 clinical queries were finalized as consensus items. The queries for the expert survey are presented in **Table 1**. The detailed Delphi procedure and expert demographic information are provided in **Supplementary Table 1**.

**Table 1:** Survey questions distributed to cardio-oncology and pharmacy experts.

No.	Survey questions	Corresponding recommendation(s)	Evidence level	Strength
1	What are the clinical values of establishing a cardio-oncology physician-pharmacist collaborative clinic?	Recommendation 1	2b	Strong
2	Which patient populations are suitable for medication therapy management in the cardio-oncology physician-pharmacist collaborative clinic?	Recommendation 6	5	Strong
3	Is clinical medication therapy management implemented in the field of cardio-oncology in your hospital?	(Background/Survey item, no specific clinical recommendation)	N/A	N/A
4	What relevant patient information should be collected in the cardio-oncology physician-pharmacist collaborative clinic?	Recommendation 7	5	Strong
5	How to conduct the efficacy evaluation of medication therapy in cardio-oncology?	Recommendation 8	5	Strong
6	How to carry out individualized adjustment of medication regimen for patients with cardio-oncology diseases?	Recommendation 9	2c	Strong
7	What types of clinical diagnosis and treatment plans should be formulated in the medication therapy management of cardio-oncology?	Recommendations 8, 9	5,2c	Strong
8	What are the contents of patient education related to medication therapy for cardio-oncology?	Addressed in Section 2.4.5 and Recommendation 10	5	Strong
9	What are the contents of follow-up management related to medication therapy for patients with cardio-oncology diseases?	Recommendations 11,12	2c,5	Strong
10	What are the requirements for personnel qualifications, clinic space and facilities in the cardio-oncology physician-pharmacist collaborative clinic?	Recommendations 2, 3, 4, 5	5	Strong
11	What are the clinical medication therapy regimens for cardio-oncology diseases?	Addressed in Section 2.4.6 (Treatment of Cardiotoxicity) and Recommendations 11,12	2c	Strong

Question 3 was a situational survey question used during Delphi rounds to understand current practice landscapes; it does not generate a prescriptive clinical recommendation.

#### 2.3.2 Evidence retrieval and determination of recommendations

Evidence Retrieval: The search incorporated both Chinese and English keywords, including “tumor”, “cancer”, “oncology”, “cardio - oncology”, “cardiac toxicity”, “cardiovascular toxicity”, “pharmaceutical clinic”, “pharmacist - managed clinic”, and “combined physician - pharmacist clinic”. Databases such as CNKI, VIP, Wanfang, PubMed, Embase, Web of Science, and international guideline websites were systematically searched. An example search strategy (PubMed) is as follows: (“Cardio -oncology”[Mesh] OR “cardiotoxicity”[tiab] OR “cardiovascular toxicity”[tiab]) AND (“Pharmaceutical Services”[Mesh] OR “Pharmacists”[Mesh] OR “pharmacist - managed clinic”[tiab] OR “physician - pharmacist clinic”[tiab]) AND (“Guideline”[ptyp] OR “Consensus”[tiab] OR “Systematic Review”[ptyp]). The retrieved evidence consisted of independently published literature, domestic and international guidelines, and national policy documents.

Evidence Quality Assessment: The evidence grading standards formulated by the Oxford Centre for Evidence - Based Medicine were employed to classify the evidence.

Inclusion/Exclusion Criteria: The following statement has been appended to Section 1.3.2:

Inclusion criteria comprised: (1) Guidelines or consensus statements on cardio-oncology management; (2) Studies evaluating pharmacist-led or collaborative cardio-oncology services; (3) Systematic reviews on cardiotoxicity prevention. Exclusion criteria included: (1) Single case reports; (2) Non-English/Chinese literature; (3) Studies focusing solely on non-pharmacological interventions without pharmacist involvement.

Recommendation Strength Grading: Consensus on recommendation strength was achieved using a 5-point scoring system. A Strong Recommendation is indicated by a mean score of 4 or higher, with at least 75% of experts rating it as a 4 or 5. A Weak Recommendation is indicated by a mean score between 3 and 4, with 50% to 74% of experts rating it as a 4 or 5. A Not Recommended designation is given when the mean score is less than 3, or when the mean score is 3 or higher but less than 50% of experts rate it as a 4 or 5.”

In accordance with the Oxford Centre for Evidence-Based Medicine framework and best practices for expert consensus development, a ‘Strong Recommendation’ may be issued even when the evidence level is low (e.g., Level 5) if the expert panel determines that the intervention represents a clear standard of care or that the clinical benefit-to-harm ratio is overwhelmingly favorable. In this consensus, recommendations regarding clinic infrastructure (e.g., Requirement for HIS access) and pharmacist qualifications were graded as Strong despite Level 5 evidence because they are foundational to patient safety and mandated by national healthcare regulations in China. The voting process during the Delphi rounds confirmed unanimous or near-unanimous agreement on the critical importance of these items.

## 3 Management of combined cardio-oncology clinics

### 3.1 Value of clinic establishment

The establishment of combined clinics, which concentrate on individualized drug therapy, aims to enhance safety, efficacy, economy, and patient adherence to treatment (9–12). The combined cardio-oncology clinic not only provides the services of a general pharmaceutical clinic but also has a specialized professional focus. It is primarily tasked with managing cardiovascular toxicity resulting from anticancer therapy or addressing pre-existing cardiac conditions to mitigate the shared risks of cancer and cardiovascular diseases. This includes providing continuous follow-up, monitoring, assessment, and medication guidance (13–15). A retrospective study indicated that pharmacist involvement in the management of cancer patients significantly reduced the incidence of adverse drug events and the average medical costs per cycle (P < 0.05), thereby improving safety and economy (16).

Recommendation 1: The combined cardio-oncology clinic offers individualized diagnosis and treatment for cardio-oncology patients, enhancing medication effectiveness, safety, adherence, and economy. (Evidence level: 2b; Strength: Strong recommendation).

### 3.2 Pharmacist qualifications, training, and assessment

#### 3.2.1 Pharmacist qualifications

There is no universal standard for pharmacist qualifications in pharmaceutical clinics, and staffing levels differ based on hospital size. Typically, it is expected that clinic pharmacists undergo standardized clinical pharmacist training (17, 18).

Recommendation 2: Pharmacists practicing in these clinics must hold a nationally recognized Certificate of Clinical Pharmacist Training. Additionally, they are required to have completed specialized training in either oncology pharmacy or cardiovascular pharmacy. Candidates must meet one of the following criteria regarding professional titles and experience:1.Hold the title of Pharmacist-in-Charge (Intermediate Level) and possess more than 5 years of clinical pharmacy experience in oncology or cardiology (or have completed relevant clinical pharmacist training);2.Hold the title of Associate Chief Pharmacist or higher, with more than 3 years of clinical pharmacy experience in oncology or cardiology. Pharmacists are encouraged to actively pursue authoritative certifications, such as the Board Certified Oncology Pharmacist (BCOP) credential from the Board of Pharmacy Specialties (BPS), and to acquire in-depth interdisciplinary training.

Leading international programs have established specialized training pathways in cardio-oncology. These typically require a Doctor of Pharmacy (Pharm.D.) degree, completion of a PGY-1 residency, and a PGY-2 residency in oncology, supplemented by reinforced cardiovascular training in practice. (Evidence level: 5; Strength: Strong recommendation).

#### 3.2.2 Pharmacist training and assessment

Pharmacists allocated to the clinic are required to undergo pre - service training and acquire clinic qualifications. Annual retraining and assessment are necessary; in cases where pharmacists fail the assessment, their qualifications shall be suspended (10). The training content comprises clinical diagnostics, pharmacotherapy, and relevant regulations (17, 18).

Recommendation 3: The training and assessment for pharmacists should cover the following domains:

1. Familiarization with regulations such as the Measures for the Clinical Application of Anticancer Drugs, the Chinese Guidelines for the Diagnosis and Treatment of Heart Failure 2024, the Principles for the Use of Novel Anticancer Drugs, and the Pharmaceutical Administration Regulations of Healthcare Institutions.
2. Mastery of theoretical knowledge in cardio - oncology, clinical laboratory diagnostics, principles of anticancer and cardiovascular drug application, and the latest domestic and international diagnostic and treatment guidelines. Newly recruited pharmacists are required to complete phased training and assessment prior to commencing their duties. The pharmacy department should conduct periodic reviews and re - assessments of the pharmaceutical services provided by clinic pharmacists. (Evidence level: 5; Strength: Strong recommendation)

### 3.3 Clinic space and equipment requirements

#### 3.3.1 Space requirements

The pharmaceutical clinic workspace ought to comply with general outpatient standards. The combined clinic shares consultation rooms with physicians (18).

Recommendation 4: Healthcare institutions are advised to establish a dedicated, shared consultation space for the combined cardio - oncology clinic within the outpatient area, with pre - scheduled clinic hours. The room should guarantee privacy. (Evidence level: 5; Strength: Strong recommendation).

#### 3.3.2 Equipment and facility requirements

The clinic’s computer should have the Hospital Information System installed (8) and be furnished with patient education materials, diagnostic flowcharts, reference books, professional literature databases, and other medical instruments. It is recommended to establish an informatized workstation for electronic documentation and implement information security and confidentiality procedures (18).

Recommendation 5: The clinic should make use of the healthcare institution’s information management system to allow pharmacists to access patient diagnoses, laboratory results, medication information, and other relevant records. Moreover, it should be equipped with professional reference books, literature databases, medication education materials, teaching aids, and relevant regulations. (Evidence level: 5; Strength: Strong recommendation)

### 3.4 Operational model of the combined clinic

#### 3.4.1 Target population

The 2022 Guidelines on Cardio-Oncology of the European Society of Cardiology (ESC) suggest that cardiotoxicity associated with anticancer drugs includes cancer therapy - related cardiac dysfunction, coronary artery disease, valvular heart disease, arrhythmias, hypertension, and other conditions (19). The 2023 guidelines of the Chinese Society of Clinical Oncology (CSCO) define cardio - oncology as the cardiovascular toxicity induced by anticancer therapy, along with pre - existing cardiovascular diseases in cancer patients (20). Based on these definitions and clinical experience, the target population for clinical practice is summarized.

Recommendation 6: The clinic offers pharmacotherapy management for:

1. Cancer patients with pre-existing cardiovascular diseases, such as coronary artery disease, arrhythmia, hypertension, dyslipidemia, thromboembolic disease, and heart failure of various etiologies, who are at risk.
2. Those utilizing anticancer drugs that may cause cardiovascular toxicity.
3. Those who have developed cardiotoxicity as a result of anticancer therapy.

Owing to the therapeutic dilemma between continuing high - efficacy anti - cancer therapy at the risk of cardiac injury and reducing dosages or discontinuing medication to protect the heart—a conflict where withdrawal may lead to tumor recurrence or progression, while continuation may result in irreversible cardiac damage—patients suffering from anti - cancer therapy - induced cardiotoxicity require increased attention. Therefore, regular outpatient follow - up is strongly recommended for this patient population. (Evidence level: 5; Strength: Strong recommendation)

#### 3.4.2 Patient information collection

For newly enrolled outpatients, a comprehensive medical history should be taken, including details of the current illness, past medical history, personal history, medication history, family history, food and drug allergies, and history of adverse drug reactions. Information on medication use and cardiac function test results (e.g., electrocardiogram [ECG], cardiac biomarkers, enzyme profiles, cardiac magnetic resonance imaging [MRI]) should be collected, and a medical record should be established (9, 13). For patients in follow - up, any changes should be recorded and archived (11).

Recommendation 7: Clinical pharmacists should collect patient information, which includes basic details, personal history, medical history, past medical history, history of adverse drug reactions, results of auxiliary examinations, and follow - up data. (Evidence level: 5; Strength: Strong recommendation)

#### 3.4.3 Treatment plan evaluation

The physician undertakes a comprehensive evaluation in accordance with the patient’s chief complaint, orders relevant examinations, and prescribes medications, thereby formulating a treatment protocol. The pharmacist assesses all medications, including past, current, and planned ones, based on evidence and the patient’s specific circumstances, evaluating the rationality of the pharmacotherapy plan in consideration of tumor status, cardiac function test results, and cardiovascular toxicity risk stratification. The effectiveness assessment entails evaluating drug resistance and the appropriateness of drug type, dosage, frequency, and duration, based on cardiac function and tumor response criteria. The safety assessment involves the management of adverse events of all therapeutic drugs, including the assessment of actual or potential cardiovascular adverse events caused by anticancer drugs, cardiotoxicity risks of combined therapy, and risks in special populations. Adherence assessment should utilize appropriate scales to comprehensively evaluate adherence and provide targeted education.

Recommendation 8: Physicians should conduct patient assessments and formulate treatment plans. Pharmacists are accountable for analyzing and evaluating these plans in terms of indications, effectiveness, safety, and adherence. The recommendations should be based on, but not restricted to, evidence, with an emphasis on the patient’s requirements for individualized therapy. (Evidence level: 5; Strength: Strong recommendation)

#### 3.4.4 Treatment plan adjustment

Following in - depth discussions and collaborative evaluations by pharmacists and physicians, adjustments are carried out, mainly encompassing medication reconciliation, deprescribing, and optimization (9, 18). Suggestions involve modifying the drug selection, dosage, or administration route of regimens that induce or may induce cardiac injury, selecting cardioprotective drugs or preventive measures, and providing usage guidance (21). A prospective study on cancer patients indicated that pharmacist intervention enhanced treatment outcomes and reduced adverse events (22).

Recommendation 9: In the combined cardio - oncology physician - pharmacist clinic, pharmacists and physicians conduct a comprehensive assessment of the patient’s cardiac function and cardiovascular disease risk after thorough consultations. When necessary, they adjust the antineoplastic regimen and implement pharmacological prophylaxis and treatment for cancer therapy - related cardiovascular toxicity. (Evidence level: 2c; Strength: Strong recommendation)

#### 3.4.5 Treatment monitoring plan

The monitoring plan primarily covers drug safety and effectiveness (11), determined based on cardiac function, cardiovascular risk, oncological status, adverse drug reactions, and interactions. In addition to conventional chemotherapy monitoring requirements for cardiovascular toxicity, more targeted agents will also be monitored for specific toxicities associated with individual agents or class of agents. For instance, androgen deprivation therapy (ADT) has been linked to arrhythmias and accelerated atherosclerosis. Thus, monitoring for such therapies should include baseline and periodic ECG assessment as well as aggressive management of metabolic syndrome panels (lipid panels, glucose monitoring). Furthermore, given the complex pharmacokinetics of many anticancer agents, monitoring will also focus on metabolic interactions (e.g., CYP450 pathways) that could impact drug exposure (AUC) and impose untoward harm, such as those seen with Bruton’s Tyrosine Kinase (BTK) Inhibitors, in addition to managing risks like QTc prolongation.

The medication guidance plan includes:

1. The potential cardiotoxicity of anticancer therapy and strategies for its management.
2. The usage, dosage, and precautions for cardioprotective drugs or those used in managing cardiotoxicity.
3. The monitoring requirements for drugs with potential cardiovascular toxicity, including the timing, frequency, and parameters.

Recommendation 10: The combined cardio-Oncology physician-pharmacist clinic should establish medication therapy monitoring plans and medication usage guidance plans for patients. Monitoring content includes: Prior to the initiation of therapy with risk of cardiotoxicity, a comprehensive baseline assessment is recommended. This should include a cardiovascular physical examination (specifically cardiac auscultation), electrocardiogram (ECG), measurement of Left Ventricular Ejection Fraction (LVEF), testing of cardiac biomarkers (such as troponin, BNP/NT-proBNP), and screening for conventional cardiovascular risk factors such as blood pressure and lipids. During treatment, once cardiotoxicity is suspected or clinical manifestations appear, one or more of the following examinations should be performed promptly based on clinical needs to confirm the diagnosis and assess cardiac function: echocardiography, serial monitoring of cardiac biomarkers, radionuclide ventriculography, cardiac magnetic resonance imaging (MRI), serial ECGs or continuous ECG monitoring, and endomyocardial biopsy. Following the completion of treatment, regular echocardiographic examinations are recommended to achieve long-term monitoring and follow-up of cardiac function. Monitoring protocols should be tailored to the specific cardiotoxicity profile of the anticancer agent.(Evidence level: 5; Strength: Strong recommendation).

#### 3.4.6 Follow-up, management, and treatment of cardiotoxicity

**Figure 1:**
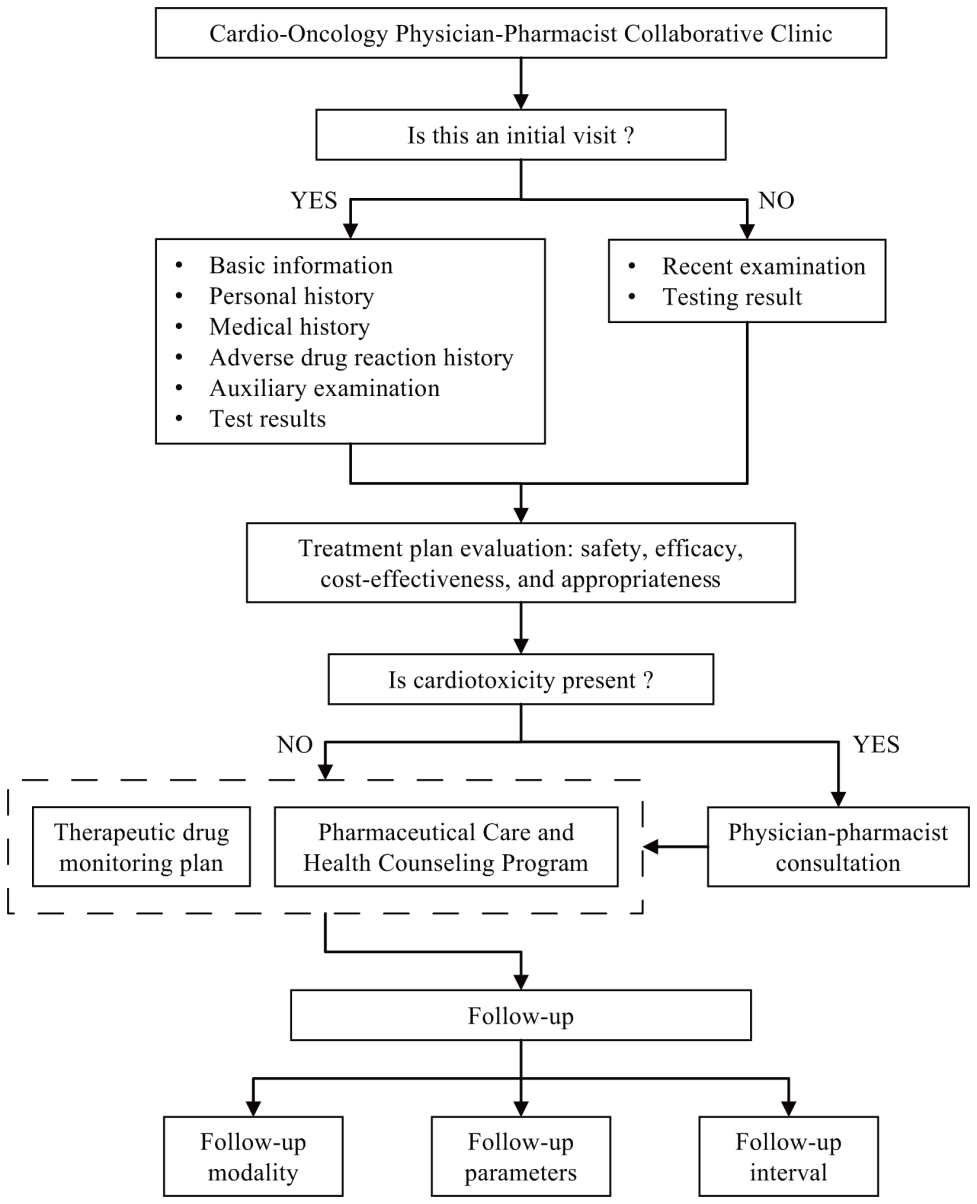
Workflow of the cardio-oncology physician-pharmacist collaborative clinic.

Follow - up in the cardio-oncology combined clinic encompasses the methods, content, and frequency of follow - up (18). Patients are followed up through telephone, online platforms, and outpatient visits. Medical institutions with adequate resources can establish electronic follow - up systems. The follow - up content comprises the safety, effectiveness, and medication compliance of pharmacological treatments, with special emphasis on the cardiovascular toxicity of antineoplastic agents. Guidelines suggest cardiac function assessment screening for asymptomatic patients with normal cardiac function 6 to 12 months after antineoplastic treatment (20). For asymptomatic very high - risk and early high - risk patients, echocardiography is recommended at 1, 3, and 5 years after antineoplastic therapy to monitor cardiac function. Follow - up investigations have demonstrated that patients in cardio-oncology combined clinics exhibit higher medication compliance and a lower incidence of adverse reactions (23). The specific workflow of the cardio-oncology combined clinic is presented in **Figure 1**.

All tumor patients should receive systematic cardiac health education and risk assessment upon discharge after completing treatment, including lifestyle guidance (e.g., diet and exercise) and necessary cardiac function screening. Research indicates that regular, moderate - intensity exercise contributes to the improvement of patients’ physical function, enhances treatment tolerance, and exerts a protective effect on the cardiovascular system (24, 25). Patients undergoing long - term antineoplastic therapy are recommended to engage in at least 150 minutes of moderate - intensity exercise per week, provided they can tolerate it (26).

At present, high - level evidence concerning specific treatment regimens for heart failure directly induced by antineoplastic drugs is scarce. Consequently, it is generally advisable for clinical management to adhere to authoritative guidelines for the diagnosis and treatment of acute and chronic heart failure. According to these guidelines, the initiation of heart failure treatment is recommended for patients with any symptomatic cardiac dysfunction caused by anthracyclines or those with symptomatic moderate - to - severe cardiac dysfunction and a left ventricular ejection fraction (LVEF) below 50% during anti - HER - 2 targeted therapy (19). In terms of pharmacological selection, guidelines advocate standard treatment regimens composed of four major drug classes: mineralocorticoid receptor antagonists, ACEIs/ARBs/angiotensin receptor - neprilysin inhibitors (ARNI), beta - blockers, and sodium - glucose cotransporter 2 inhibitors (SGLT - 2i) (27, 28). These agents should serve as the cornerstone of heart failure therapy.

Recommendation 11: Formulate individualized follow - up plans based on patient status, including:

1. Follow - up approaches: Pharmacists in the cardio-oncology combined clinic conduct regular follow - ups through telephone, online platforms, and outpatient visits.
2. Follow - up content: Assessment of medication treatment goals, identification of new medication - related issues, occurrence of adverse drug events, medication compliance, and tracking of examination results.
3. Follow - up frequency: Pharmacists in the cardio-oncology combined clinic should conduct regular follow - ups as necessary. Patients with special disease conditions require intensified follow - up. Asymptomatic patients with normal cardiac function should undergo cardiac function assessment screening, including LVEF and myocardial injury markers, within 6 to 12 months after receiving antineoplastic therapy with potential cardiotoxic effects, followed by regular screening two years post - treatment. Asymptomatic very high - risk and early high - risk patients should undergo echocardiography at 1, 3, and 5 years after antineoplastic therapy to monitor cardiac function. Before initiating cardiotoxic antineoplastic drugs, a comprehensive risk assessment should be carried out, including cardiovascular physical examination and baseline cardiac evaluations. Patients with an LVEF below 50% after antineoplastic therapy are recommended to initiate cardioprotective therapy. For heart failure induced by antineoplastic drugs, mineralocorticoid receptor antagonists, ACEIs/ARBs/ARNI, beta - blockers, and SGLT - 2 inhibitors are recommended to improve long - term prognosis. (Quality of evidence: 2c; Strength of recommendation: Strong)

#### 3.4.7 Long-term cardiovascular monitoring and management during survivorship

Patients entering the survivorship phase after the completion of cancer therapy remain at risk for late-onset cardiotoxicity. In clinical practice, physicians and pharmacists in the cardio-oncology joint clinic should jointly develop a long-term monitoring plan and utilize the Hospital Information System (HIS) to remind patients of follow-up visits to avoid loss to follow-up. During this phase, pharmacists need to focus specifically on patient adherence to long-term medication (e.g., ACEIs/β-blockers) and provide lifestyle counseling to achieve lifelong protection of cardiac function.

Recommendation 12:For cancer survivors who have received potentially cardiotoxic anti-tumor therapy, the cardio-oncology joint clinic should assume primary responsibility for long-term survivorship monitoring, rather than referring them to general survivorship clinics. It is essential to ensure that echocardiographic follow-up is completed at 1, 3, and 5 years post-treatment in accordance with guideline requirements, with interpretation and intervention provided by a specialist team (Quality of Evidence: 5a; Strength of Recommendation: Strong Recommendation).

#### 3.4.8 Delineation of roles in the combined clinic

To ensure efficient and safe practice, the responsibilities within the combined clinic are delineated as follows:

Pharmacist-Led Tasks:

1. Comprehensive medication reconciliation (including over-the-counter drugs and supplements).
2. Screening for potential drug-drug interactions, particularly metabolic interactions that can significantly impact drug concentrations (AUC exposure) and impose untoward harm, including but not limited to QTc prolongation, arrhythmia risks (e.g., atrial fibrillation, ventricular tachycardia with BTK inhibitors), and other cardiotoxic effects.
3. Patient education on medication adherence and adverse effect self-monitoring.

Physician-Led Tasks:

1. Clinical diagnosis of cardiovascular complications.
2. Ordering and interpretation of advanced imaging (e.g., Cardiac MRI, ECHO).
3. Primary prescribing of anticancer and cardioprotective agents.

Joint Decision-Making Tasks:

1. Therapeutic Dilemma Resolution: Deciding between temporary interruption vs. continuation of cardiotoxic anticancer therapy when LVEF declines or troponin elevates.
2. Risk-Benefit Reassessment: Balancing oncologic efficacy against cardiovascular safety in high-risk patients.
3. Deprescribing: Determining when to taper or discontinue cardiovascular medications in stable survivors.

## 4 Conclusion

This consensus document puts forward a series of standards and recommendations for the management of integrated cardio - oncology physician - pharmacist clinics. The publication of these guidelines is intended to standardize the management procedures of the clinics, delineate the qualifications and assessment criteria for pharmacists, guarantee the availability of appropriate facilities and equipment, and establish scientific workflows. The objective is to enhance medication efficacy, safety, adherence, and cost - effectiveness, thus ultimately safeguarding the quality of life of patients. It is expected that the implementation of this consensus will elevate the level of pharmaceutical services in combined cardio - oncology clinics, providing more professional and comprehensive diagnostic and management services to cancer patients.
