# Supplementary material for: Expert consensus from the Chinese medical association on pharmaceutical management of combined cardio-oncology physician-pharmacist clinics
Source: Front Oncol. 2026 Jun 17;16:1741387. doi: 10.3389/fonc.2026.1741387 (PMC13318725; doi:10.3389/fonc.2026.1741387)
Supplement: Supplementary file 1 [file Table1.docx]

| NO. | Survey Questions | Average score | Full score ratio | Coefficient of Variation | Total Level | Score proportion | Retention or Removal |
| --- | --- | --- | --- | --- | --- | --- | --- |
| 1 | What are the clinical values of establishing a cardio-oncology physician-pharmacist collaborative clinic? | 4.67 | 72.22% | 0.127 | 84 | 93.33% | Retention |
| 2 | Which patient populations are suitable for medication therapy management in the cardio-oncology physician-pharmacist collaborative clinic? | 4.83 | 88.89% | 0.106 | 87 | 96.67% | Retention |
| 3 | Is clinical medication therapy management implemented in the field of cardio-oncology in your hospital? | 4.50 | 66.67% | 0.190 | 81 | 90.00% | Retention |
| 4 | What relevant patient information should be collected in the cardio-oncology physician-pharmacist collaborative clinic? | 4.50 | 66.67% | 0.190 | 81 | 90.00% | Retention |
| 5 | How to conduct the efficacy evaluation of medication therapy in cardio-oncology? | 4.33 | 61.11% | 0.209 | 78 | 86.67% | Retention |
| 6 | How to carry out individualized adjustment of medication regimen for patients with cardio-oncology diseases? | 4.39 | 55.56% | 0.177 | 79 | 87.78% | Retention |
| 7 | What types of clinical diagnosis and treatment plans should be formulated in the medication therapy management of cardio-oncology? | 4.44 | 66.67% | 0.207 | 80 | 88.89% | Retention |
| 8 | What are the contents of patient education related to medication therapy for cardio-oncology? | 4.72 | 77.78% | 0.121 | 85 | 94.44% | Retention |
| 9 | What are the contents of follow-up management related to medication therapy for patients with cardio-oncology diseases? | 4.33 | 50% | 0.177 | 78 | 86.67% | Retention |
| 10 | What are the requirements for personnel qualifications, clinic space and facilities in the cardio-oncology physician-pharmacist collaborative clinic? | 4.28 | 55.56% | 0.224 | 77 | 85.56% | Retention |
| 11 | What are the clinical medication therapy regimens for cardio-oncology diseases? | 3.94 | 54.44% | 0.295 | 71 | 88.89% | Retention |

Supplementary Table S1. The detailed Delphi process and expert demographics
